# Emerging investigator series: metagenomic insights into microbial controls of carbon cycling in alpine soils

**DOI:** 10.1039/d5em01047k

**Published:** 2026-04-28

**Authors:** Kristina Bright, Bence Dienes, Bart van Dongen, Ilya Strashnov, Xingguo Han, Meret Aeppli

**Affiliations:** a Soil Biogeochemistry Laboratory (SOIL), Swiss Federal Institute of Technology Lausanne (EPFL) Sion Switzerland meret.aeppli@epfl.ch +41 21 693 72 79; b Department of Earth and Environmental Sciences, University of Manchester Manchester UK; c Swiss Federal Institute for Forest, Snow and Landscape Research (WSL) Birmensdorf Switzerland

## Abstract

Alpine riparian zones span topographic gradients from wet soils on the plain near streams to drier soils on adjacent slopes. These differences in soil moisture are generally associated with shifts in the soil redox state from anoxic on the plain to oxic on the slope. In anoxic plain soils, soil organic carbon (SOC) may accumulate due to thermodynamic constraints on microbial activity. Here, we used shotgun metagenomics to examine how microbial diversity and functional potential vary across differing redox conditions on plain and slope soils in two catchments in the Swiss Alps. We complemented these analyses with soil physicochemical characteristics and information on the chemical composition of organic matter. Plain soils had higher SOC stocks and higher relative abundance of phenol compounds relative to slope soils, consistent with SOC preservation and preferential mineralisation of easily degradable organic compounds under anoxic conditions. Microbial communities in plain soils further exhibited greater taxonomic and functional diversity, including increased potential for anaerobic respiration pathways. Genes for nitrate, iron, and sulfate reduction were linked to the *Chloroflexota*, *Acidobacteria*, and *Desulfobacterota* phyla, respectively. Based on NMDS correlations, electron accepting capacity, calcium content, and pH shaped microbial community composition. Slope soils, by contrast, supported less diverse microbial communities, determined mainly by electron donating capacity and clay content. Our work demonstrates how soil redox conditions and microbial functional potential shape carbon cycling across landscape positions in alpine riparian zones. This mechanistic understanding is critical to anticipate changes in carbon cycling in alpine ecosystems in a changing climate.

## Abstract 2

In alpine riparian zones, plain soils contained more organic carbon, were more reduced, and had higher microbial diversity than slope soils.

## Graphical abstract

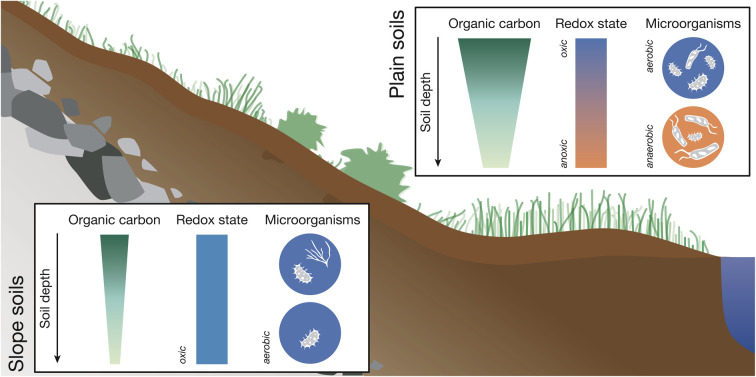

**Environmental significance**

Redox conditions in alpine riparian soils regulate whether soil organic carbon is preserved or mineralized. Electron-acceptor availability shifts over short topographic distances – from wet, anoxic plains, to drier, oxic slopes – driving corresponding changes in microbial metabolism. We paired shotgun metagenomics with analyses of the soil redox state and organic matter composition to trace how functional gene inventories align with soil redox regimes. Plain soils held more soil organic carbon, were more reduced and showed higher microbial taxonomic and functional diversity, with enriched capacities for nitrate, iron, and sulfate reduction compared to slope soils. Phenolic compounds were relatively enriched in anoxic plain soils, consistent with thermodynamic preservation of soil organic matter. Our findings help anticipate how alpine carbon cycling may shift in a warming climate.

## Introduction

In subalpine and alpine ecosystems, more than 90% of ecosystem carbon is stored in soils as a consequence of short plant growing seasons and limitations on the degradation of soil organic matter by microorganisms under harsh climatic conditions.^1,2^ The fate of organic carbon in the soil is determined by microorganisms that can mineralize organic matter to greenhouse gases or stabilize it within soils.^3,4^ It remains unclear how SOC stocks are linked to microbial community composition and functional potential in (sub)alpine ecosystems.

Alpine riparian zones express strong differences in hydrology and soil biogeochemistry between soils on low-lying plains near streams to those on adjacent slopes.^5,6^ Plain soils, influenced by shallow groundwater and seasonal water inputs, are periodically saturated, producing oxygen-limited redox conditions where microbial respiration depends on alternative terminal electron acceptors (TEAs). These less energy-efficient pathways slow organic matter decomposition and promote SOC accumulation.^7–10^ In contrast, slope soils are well drained and maintain more oxidized conditions that support aerobic microbial activity and greater SOC mineralization, resulting in smaller SOC stocks relative to plains.^11^

Soil redox conditions, along with other edaphic factors such as pH and nutrient availability, are linked to microbial community composition and metabolic diversity.^12^ Microbial characteristics can be assessed using metagenomics, which has proven particularly valuable in extreme environments such as thawing permafrost, where genomic analyses have revealed microbial adaptations to redox-stratified conditions and geochemical gradients,^13–15^. Ref. 13, for instance, showed that microbial iron reduction strongly influences microbial carbon degradation in thawing permafrost. Similarly, ref. 14 demonstrated that permafrost microbial communities and functional genes are structured by latitudinal gradients and soil geochemistry. In alpine plains, fluctuating water tables and variable oxygen conditions likely necessitate a wide microbial metabolic repertoire, enabling microorganisms to adapt their respiration strategies to the availability of TEAs.^10,16,17^ Alpine systems therefore express similar redox variability and potentially microbial adaptation strategies as thawing permafrost, yet integrated metagenomic assessments remain rare in alpine environments.

Here, we investigate the relationships between SOC stocks, microbial community structure and functional potential, and environmental conditions across plain and slope areas in two alpine headwater catchments. Although environmental conditions differ slightly between the two catchments, both share similar geomorphic structures and hydrological regimes and can therefore be treated as landscape-level replicates. We hypothesise that

1. Plain soils exhibit anoxic conditions that are associated with higher SOC contents and higher levels of poorly degradable SOC, such as phenols and aromatics.

2. Microbial communities exhibit greater metabolic diversity in plain soils than in slope soils, driven by the larger temporal variability in soil redox conditions.

To test our hypotheses, we combined the analysis of soil physicochemical characteristics with analyses of the soil redox state by mediated electrochemistry, soil organic matter chemistry by pyrolysis gas chromatography-mass spectrometry, and microbial functional diversity and metabolic capabilities by shotgun metagenomics. We compared SOC content and composition across landscape positions and soil depths, correlated taxonomic lineages with functional gene potentials, and incorporated environmental vectors into a non-metric multidimensional scaling (NMDS) ordination analysis to assess relationships between microbial communities and environmental factors.

## Materials and methods

### Site description and sample collection

**Fig. 1:**
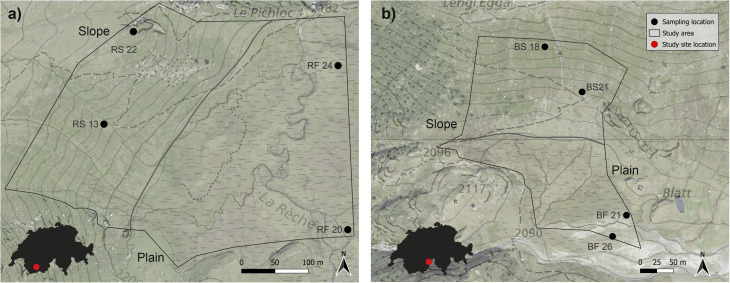
Sampling locations in (a) Réchy and (b) Binntal showing the spatial layout of plain and slope sites across the two catchments. The map is intended to provide site context; depth-specific SOC differences are presented separately in Fig. 2. Locations where the 30–50 cm interval was unavailable are indicated in Table S1.

Soils were collected from the riparian areas of two natural headwater catchments in the Swiss Alps: Blatt in the Binntal valley (46°22′N/8°16′E) and Ar du Tsan (46°12′N/7°30′E) in the Vallon de Réchy. Both catchments feature a mixed bedrock mainly composed of gneiss and carbonated rock (Matteodo *et al.*, 2018;^18^ swisstopo, 2024^19^). They are characterised by siliceous alpine grasslands and moorlands. Vegetation differs systematically between slope and plain positions. Slope areas are dominated by subalpine acidophilous grassland and heath communities, whereas plain areas support wetland vegetation typical of alkaline and acidophilic fens. Reported plant community types include *Nardion*, *Rhododendro-Vaccinion*, and *Loiseleurio-Vaccinion* on slopes and *Caricion davallianae*, *Caricion fuscae*, *Calthion*, and *Caricetum rostratae* on plains.^20–22^ The sampling sites within the two catchments were strategically chosen to encompass both slope and plain areas. At Réchy, the elevation of sampling locations varied from 2154 to 2243 meters above sea level (m a.s.l.), while at Binntal, the range was between 1984 and 2105 m a.s.l. Soil sampling was carried out in late July 2023. Average July temperatures at l’Ar du Tsan and Binntal are 12.9 °C and 8.7 °C, with precipitation levels of 76 mm and 97 mm, respectively.^23^ At 8 sampling locations (Fig. 1 and Table S1), soil from three soil depths (0–10 cm, 10–30 cm, and 30–50 cm, when available) was collected with an auger. Precise coordinates, slope, and specific landscape position were recorded for each location. Once gathered, the samples were placed in zip-lock bags; bags for water-logged soils contained oxygen scrubbers. Samples were kept cool with ice packs and transported to the laboratory. Samples for soil physicochemical characterization were immediately processed; sub-samples for soil redox characterization were stored at −20 °C until analysis. Samples for DNA extraction were placed in a sterile manner into Whirl-Pack® bags and homogenised directly by kneading. Post-homogenisation, the soil was split into triplicate subsamples, placed into cryotubes and shock-frozen using liquid nitrogen. After transport to the laboratory, they were stored at −80 °C until analysis.

### Soil physicochemical characterisation

#### Sample preparation, pH measurements, and soil texture analyses

Soil samples were air-dried and subsequently oven-dried at 105 °C, homogenised, sieved through a 2 mm mesh to remove coarse particles (*e.g.*, plant roots and stones), and ground using a ball mill (Pulverisette 7, Fritsch) to achieve a fine, uniform powder. Dry weight was determined from changes in soil mass upon drying. Soil pH was measured using a SevenDirect SD50 pH meter, Mettler Toledo in a 1 : 5 soil-to-deionised water suspension after 30 minutes of agitation at 200 rpm, followed by 30 minutes of settling (Table S1). Soil texture was analysed using laser diffraction (LS 13 320, Beckman Coulter) with a grain size analyser, on 0.5 g of air-dried, sieved bulk soil following organic matter digestion with hydrogen peroxide over two weeks (Table S1).

#### Elemental composition

Total carbon was measured by chromatography after combustion at 900 °C on a CHNS element analyser (Flash EA 1112, Thermo Finnigan). As the soils lacked carbonates, the total SOC content (expressed as weight % of dry soil) was considered equivalent to the measured total carbon. Total content of Fe, Mn, and S was determined on 5 g of dried, sieved, and powdered soil using X-ray fluorescence spectroscopy (SPECTRO XEPOS).

#### SOC composition

The relative abundance of major compound classes was determined using pyrolysis gas chromatography-mass spectrometry (Py-GC-MS).^24^ Soil samples were placed in clean, fire-polished quartz tubes and pyrolysed at 600 °C for 20 seconds under a helium flow. The released pyrolysis moieties were transferred *via* a heated transfer line into an Agilent 7980A GC equipped with a Zebron ZB-5MS column (Phenomenex, Woerden, the Netherlands; 30 m × 250 µm × 0.25 µm) coupled to an Agilent 5975C MSD single quadrupole mass spectrometer operating in electron ionisation mode (scanning *m*/*z* 50 to 650 at 2.7 scans per second; ionisation energy: 70 eV), using helium as the carrier gas and introduced in split mode (70 : 1 split ratio; a constant flow of 2 ml per min, with gas saver mode active). The pyrolysis transfer line and rotor oven temperature were maintained at 325 °C, the heated GC interface was maintained at 280 °C, the electron ionisation source was maintained at 230 °C, and the quadrupole was maintained at 150 °C. The GC oven was programmed from 40 °C (held for 5 minutes) to 300 °C at 5 °C per minute, where it was held for 3 minutes, giving a total run time of 60 minutes. Approximately 106 of the most abundant pyrolysis moieties were identified, by comparing their retention times and spectra to those of entries in the NIST Mass Spectral Library and grouped into categories based on their origin and chemical characteristics: lipids, lignins, polysaccharides, phenols, nitrogen containing compounds, and aromatics (Fig. S1). Given the complexity of the programs, it was not possible to integrate individual moieties in total ion current mode due to significant overlap between ion peaks. Instead, single ion filtering was used to measure the peak area of each compound. The major ions of each compound were filtered and integrated (Table S2). The relative abundance of each identified compound was calculated as a percentage of the total identified compounds.

#### Electron accepting & donating capacities

Electron accepting and electron donating capacities (EAC & EDC) were determined through mediated electrochemical analyses using an 8-channel potentiostat (CH Instruments, Inc.) in an anoxic environment inside a glovebox workstation (Labmaster pro MBraun), as previously described.^25,26^ Experiments were conducted using a pH-buffered solution at pH 5.5 (0.4 M sodium acetate–acetic acid) with 10 mM sodium chloride as a background electrolyte. Mediated electrochemical reduction (MER) potentials were set *versus* standard hydrogen electrodes at −0.51 V *vs.* SHE (*E*_H, MER_) and mediated electrochemical oxidation (MEO) potentials at +0.82 V *vs.* SHE (*E*_H, MEO_). For EAC measurements, ethyl viologen^27^ was used as an electron transfer mediator; for EDC, 2′2-azino-bis(3-ethylbenzothiazoline-6-sulfonic acid (ABTS)^28^ was used. To prepare the samples, 1 g of frozen soil was transferred into 10 mL of Milli-Q water under anaerobic conditions to create a slurry. For each measurement, 30 µL of the slurry was used for EAC determination, and 20 µL was used for EDC determination. An additional 1 mL aliquot was taken from each slurry in triplicate to determine soil dry weight for normalisation. EAC and EDC values were determined from current responses measured upon the addition of the sample to the electrochemical cells at *E*_H, MER_ and *E*_H, MEO_, respectively. Capacities (mol e^−^ per g dried soil) were determined by integrating the baseline-corrected current, *i*(*t*), over time, from *t*_0_ until the current returned to the baseline at *t*_end_ following eqn (1) and (2).1
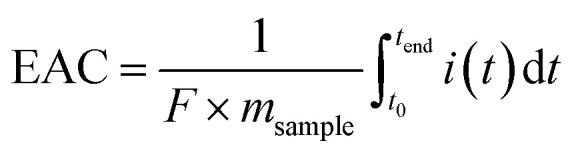
2
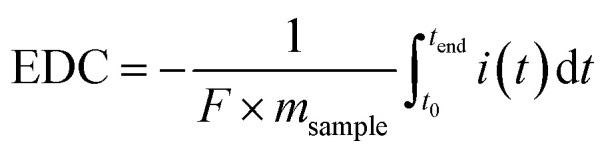
where *F* ≈ 96485 C mol^−1^ is the Faraday constant and *m*_sample_ is the dry mass of the soil.

### DNA extraction, sequencing, and analysis

DNA was extracted from 0.5 g of soil using the DNeasy PowerSoil Pro Kit (Qiagen, Germany) following the manufacturer's protocol. DNA integrity was assessed using an Agilent 5400 system. DNA content and purity were assessed using microspectrophotometry (NanoDrop One; Thermo Fisher Scientific Inc., USA). Library preparation and shotgun metagenomic sequencing were performed by Novogene (UK) with an Illumina NovaSeq 6000 platform to generate paired-end (150 bp) reads. Across the 20 samples, sequencing yielded an average of 103.7 million raw reads per sample (range: 83.7–132.1 million), corresponding to an average of 15.5 Gb raw data per sample (range: 12.6–19.8 Gb). Assembly quality was evaluated using QUAST; across 19 assemblies, the average total contig length was 673.7 Mb (range: 422.7–1094.5 Mb) and the average N50 was 979 bp (range: 783–1395 bp).

Initial quality checks of raw sequencing reads were conducted using FastQC to ensure data integrity.^29^ Reads were subjected to quality filtering using fastp,^30^ followed by a second round of quality checks with FastQC to verify improvements in read quality. *De novo* assembly of high-quality reads was performed with MEGAHIT, generating contigs suitable for downstream analyses.^31^ Assembly statistics were evaluated using QUAST to ensure completeness and accuracy.^32^ High-quality reads were mapped to assembled contigs using Strobealign to generate coverage profiles.^33^ Metagenome-assembled genomes (MAGs) were reconstructed using MetaBAT2,^34^ and bin quality was assessed using CheckM2 to ensure completeness and contamination metrics were within acceptable thresholds.^35^ Taxonomic classification of MAGs was assigned using the Genome Taxonomy Database Toolkit (GTDB-Tk).^36^ Functional annotation of MAGs was conducted using METABOLIC,^37^ allowing for the prediction of key metabolic pathways and biogeochemical functions. Dereplication of MAGs was performed using dRep to consolidate redundant genomes and generate a representative set.^38^ The relative abundance of dereplicated MAGs was calculated using CoverM.^39^

### Statistical analysis

SOC content and composition were analysed using Wilcoxon rank-sum tests for comparison of plain and slope soils and one-way ANOVA with Tukey's HSD *post-hoc* tests for soil depth effects. Spearman correlations were used to examine associations between functional gene categories and taxonomic lineages identified in the metagenomic data. NMDS analysis was used to visualise microbial community dissimilarities based on Bray–Curtis distances and identify effects of environmental factors on microbial community composition. This analysis was complemented by a PERMANOVA test to assess the influence of location, landscape position, and soil depth on microbial community structures. Figures and statistical analyses were generated in R using the vegan package to explore microbial community composition and diversity metrics.^40^

## Results

### Plain soils store more organic carbon than slope soils

**Fig. 2:**
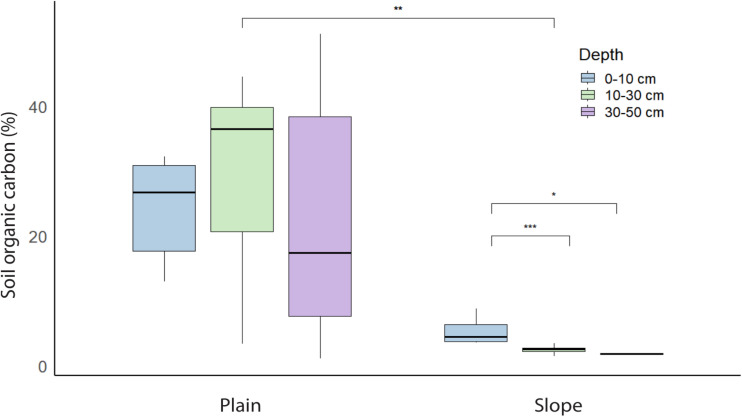
Soil organic carbon (SOC) content, expressed as percentage of dry soil mass, in plain and slope soils at three soil depths (0–10 cm, 10–30 cm, and 30–50 cm). Boxplots show the distribution of individual samples within each group; centre lines indicate medians, boxes indicate interquartile ranges, and whiskers indicate the range of the data excluding outliers. Asterisks indicate significant differences between plain and slope soils within each depth interval (*p < 0.05, **p < 0.01, and ***p < 0.001). Sample numbers vary among depth intervals because the 30–50 cm layer was not available at all sampling locations (Table S1).

Plain soils had higher SOC contents than slope soils at all soil depths (Fig. 2): the SOC range was 24.3 ± 8.3% in the 0–10 cm layer and 22.8 ± 20.5% at 30–50 cm with the highest SOC values observed in the mid-soil depth layer (10–30 cm: 29.6 ± 16.3%) for plain soils. In contrast, slope soils exhibited a clear soil depth-dependent trend in SOC concentrations, with SOC decreasing from 6.49 ± 4.92% in the surface layer (0–10 cm) to 2.48 ± 0.66% at 10–30 cm and 1.80 ± 0.20% at 30–50 cm. SOC content in plain soils was significantly higher than that in the slope across all soil depths, with mean SOC at 0–10 cm soil depth in plain soils being approximately 3.7 times higher than that in slope soils.

### Soil organic carbon composition differs by landscape position and soil depth

**Fig. 3:**
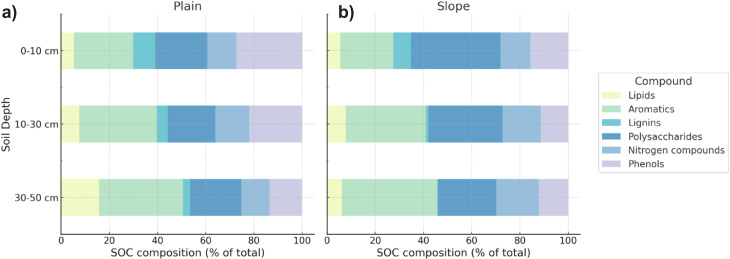
Composition of soil organic carbon (SOC) in (a) plain and (b) slope soils, shown as the relative abundance (% of total identified SOC) of major compound classes determined by pyrolysis gas chromatography-mass spectrometry (Py-GC-MS). Compound classes include lipids, aromatics, lignins, polysaccharides, nitrogen-containing compounds, and phenols. Values represent individual soil samples across all sampled depths; relative abundances for each sample are provided in Table S3.

The composition of organic matter varied by landscape position and soil depth (Fig. 3). Slope soils were enriched in polysaccharides (31.9%), whereas plain soils contained higher proportions of phenols (20.9%). The proportions of aromatics, lipids, nitrogen-containing compounds, and lignin were comparable between both types of soils. The relative contribution of aromatic compounds increased with soil depth from 23.3% at 0–10 cm to 36.4% at 30–50 cm, while the relative contribution of lignins decreased from 8.2% at 0–10 cm to 2.1% at 30–50 cm.

### Plain soils exhibit soil depth-dependent redox zonation

**Fig. 4:**
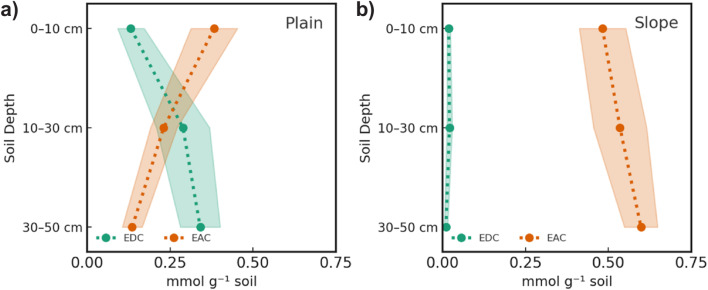
Average electron donating capacity (EDC, green) and electron accepting capacity (EAC, orange) of (a) plain and (b) slope soils across the three sampled depth intervals. Capacities are expressed as mmol electrons per gram of dry soil and were determined by mediated electrochemical oxidation and reduction at pH 5.5 and +0.82 V and −0.51 V *vs.* SHE, respectively. Shaded areas represent the standard error of the mean.

The soil redox state was described using the EAC and EDC values, which represent the contribution of the soils' pools of redox-active oxidised and reduced geochemical species, respectively. In plain soils, EAC and EDC values exhibited an inverse relationship, with EDC increasing from 0.12 ± 0.05 to 0.33 ± 0.07 mmol g^−1^ soil and EAC decreasing from 0.41 ± 0.10 to 0.16 ± 0.05 mmol g^−1^ soil from 0–10 cm to 30–50 cm soil depth, consistent with increasingly reducing conditions (Fig. 4a). In contrast, slope soils showed low EDC values (*e.g.*, 0.02 ± 0.01 at 10 cm) and constant EAC values (*e.g.*, 0.48 ± 0.12 at 10 cm) across all soil depths, indicating oxic conditions (Fig. 4b). We compared total electron exchanging capacity (sum of EAC and EDC) to elemental composition to attribute the EAC and EDC responses to geochemical phases (Fig. S2). In plain soils, iron explained most of the redox activity, followed by sulfur. In slope soils, iron was the dominant redox-active phase, with a small contribution from redox-active organic matter.

### Microbial community composition and functional potential are linked to soil redox conditions

**Fig. 5:**
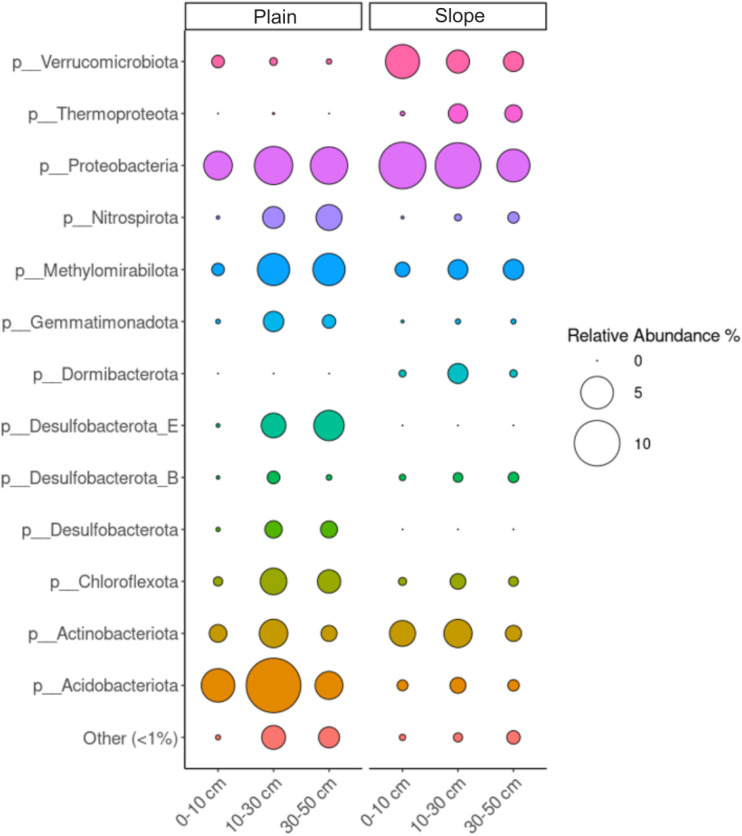
Microbial community composition in plain and slope soils across three soil depths. The plot shows the relative abundance of selected dominant prokaryotic phyla identified from metagenomic data. Circle size represents the relative abundance of each taxon within a given sample group, allowing comparison of taxonomic patterns across landscape positions and soil depths.

**Fig. 6:**
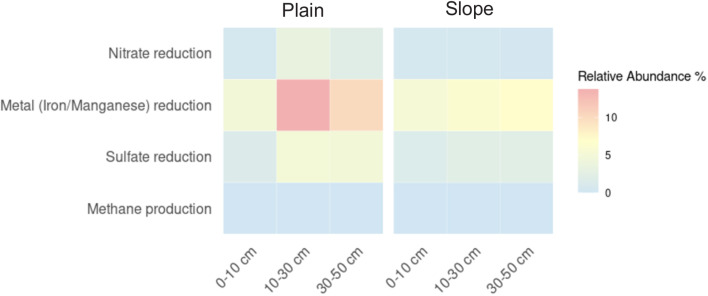
Heatmap of putative anaerobic microbial respiration pathways in plain and slope soils across the three soil depths. Colours indicate the relative abundance of genes assigned to key metabolic pathways reconstructed from metagenomic data, including nitrate reduction, metal reduction, sulfate reduction, and methane production.

Microbial community composition differed between plain and slope soils (Fig. 5): plain soils had higher relative abundances of *Chloroflexota*, *Acidobacteriota*, and *Desulfobacterota*, whereas slope soils contained greater proportions of *Verrucomicrobiota*, *Thermoproteota*, and *Dormibacterota*. The heat map of functional genes (Fig. 6) shows that plain soils exhibited higher relative abundances of genes assigned to nitrate, metal (Fe/Mn), and sulfate reduction across all soil depths, while slope soils had lower relative abundances of these genes. Canonical methanogenesis genes were not detected in the recovered MAGs, whereas genes attributed to methane oxidation (*Methylomirabilota*) were present in both landscape positions at low abundance.

Taxon–function links were identified between specific microbial lineages and key reductive processes (Table S3). *Chloroflexota* lineages (*e.g.*, class *Anaerolineae*; class *Dehalococcoidia* lineages DSTF029 and SM23-31) correlated with nitrate-reduction genes. *Acidobacteriota* classes *Thermoanaerobaculia*, *Acidobacteriae*, and *Blastocatellia* correlated with Fe/Mn-reduction genes. Orders within *Desulfobacterota* (*Geobacterales*, BSN033, and *Desulfatiales*) correlated with sulfate-reduction genes.

### Microbiomes exhibit enhanced metabolic versatility and greater potential for anaerobic carbon turnover in plain soils

**Fig. 7:**
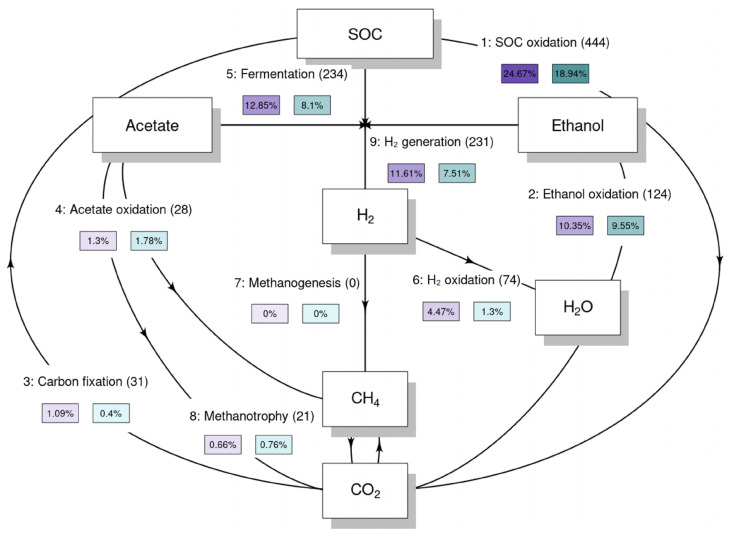
Soil organic carbon (SOC) transformations mediated by microbial communities in plain and slope soils. The flowgram illustrates SOC-related metabolic steps reconstructed from metagenomic data using a modified script from METABOLIC.^37^ Each arrow represents a distinct transformation step, with boxes denoting key compounds involved. Arrow labels indicate the step number and transformation type, the number of genomes encoding the necessary genes (in brackets), and the relative abundance of those genomes in plain (purple) and slope soil communities (teal), expressed as a percentage of total community composition. Community-level genome abundance and function were inferred from metagenome-assembled genomes.

We assessed the core set of metabolic functions relative to carbon turnover following the flowgram pipeline by ref. 37. Distribution of these functions, showing the proportion of metagenome-assembled genomes (MAGs) that encode the genes required for each transformation, indicates that SOC oxidation genes were present in 24.67% of plain soil genomes (444 MAGs) *versus* 18.94% in slope soil genomes (Fig. 7). Fermentation potential was likewise greater in plain soil communities (12.85%; 234 MAGs) than in slope soil communities (8.10%). Hydrogen generation genes occurred in 11.61% of plain soil genomes (231 MAGs) compared with 7.51% of slope soil genomes, whereas hydrogen oxidation genes were found in 4.47% and 1.30% of genomes, respectively (74 MAGs). In contrast, acetate-oxidation genes showed slightly higher representation in slope soil communities (1.78%; 28 MAGs) than in plain soil communities (1.30%) while genes for ethanol oxidation and carbon fixation were detected at low levels in both settings (ethanol oxidation: 10.35% plain and 9.55% slope; carbon fixation: 1.09% plain and 0.40% slope). Methanotrophy genes were rare but detectable (0.66% plain and 0.76% slope; 21 MAGs), whereas canonical methanogenesis genes were not detected in the recovered assemblies. Overall, the higher prevalence of fermentation, hydrogen metabolism, and SOC-oxidation genes in plain soil MAGs indicates a larger genomic investment in anaerobic carbon turnover than in slope soils.

### Microbial community composition correlates with soil physicochemical properties across landscape positions and catchments

**Fig. 8:**
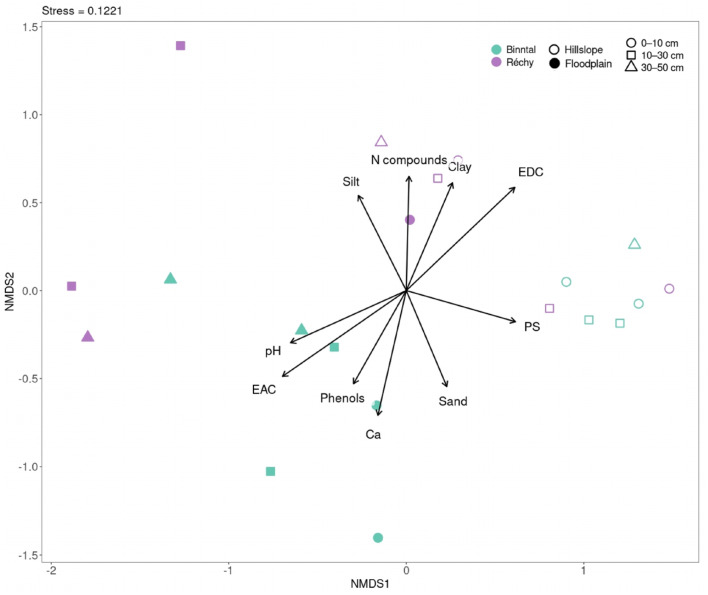
NMDS plot illustrating the microbial community composition of soils from two alpine headwater catchments, Binntal and Réchy, based on Bray–Curtis dissimilarity. Environmental vectors overlaid on the ordination indicate the direction and strength of correlations between environmental variables and microbial community composition. Vector length is scaled by the square root of the *r*^2^ value, reflecting the strength of these correlations. The vectors represent the top ten environmental variables, selected based on descending *r*^2^ values from envfit analysis. These variables include polysaccharides (PS), nitrogen compounds (N compounds), silt, clay, sand, soil pH, electron accepting capacity (EAC), calcium (Ca), phenols, and electron donating capacity (EDC). PERMANOVA attributes 23.3% of the Bray–Curtis variation to landscape position (*R*^2^ = 0.2325, *p* = 0.001) and 10.0% to catchment identity (*R*^2^ = 0.0999, *p* = 0.030); soil depth effects are negligible.

Similarities between microbial community composition across catchments and landscape positions were assessed using an NMDS plot (Fig. 8, supplementary environmental values are in Table S1). Plain soil communities cluster on the bottom left, while slope soil communities cluster on the top right with minimal overlap between groups. Vectors for Ca content, soil pH, EAC, and phenols have the largest percentage value and point toward the plain soil cluster, indicating strong positive correlations with those communities. Conversely, vectors for polysaccharides, nitrogen-containing compounds, EDC, and clay content project toward the slope soil cluster. Sand and silt vectors plot between the two groups with intermediate vector lengths. Thus, variation in Ca content, pH, EAC, EDC, and specific SOC fractions (phenols, nitrogen compounds, and polysaccharides) aligns with the primary ordination axis that separates plain and slope microbial assemblages.

## Discussion

### Soil redox state is linked to soil organic carbon quantity and chemistry

Plain soils had substantially higher SOC content than slope soils, with nearly four times more carbon per g dry soil in the surface layer (0–10 cm; Fig. 2). These differences mirrored differences in the soil redox state (Fig. 4). In plain soils, conditions became increasingly reducing with soil depth. The observed EDC values likely reflect the accumulation of reduced organic compounds, ferrous iron, and sulfide, produced *via* microbial respiration under past anoxic conditions. Iron was the major contributor to electron exchanging capacity and was therefore a key TEA in plain soils. In slope soils, no EDC was detected, suggesting that these soils were fully oxic. Most of the EAC response was explained by iron with some contribution from organic matter. The observed patterns in SOC content and the soil redox state are therefore in agreement with our first hypothesis stating that soils on the plain exhibit anoxic conditions that are associated with higher SOC contents.

Differences in SOC composition between plain and slope soils are likely due to variations in organic matter inputs and preservation mechanisms. Plain soils were enriched in phenolic compounds, whereas slope soils contained higher proportions of polysaccharides (Fig. 3). This pattern was linked to contrasting vegetation types and moisture regimes. On the plain, grasses and sedges produce litter rich in phenol-containing structural polymers, which are selectively preserved under periodically anoxic conditions because the degradation of phenolic compounds depends on extracellular oxidative enzymes, such as phenol oxidase and peroxidase.^41,42^ In contrast, slope soils dominated by dwarf shrubs and upland herbs receive litter rich in easily degradable carbohydrates, which likely causes the higher abundance of polysaccharides. Across both landscape positions, we observed a decline in lignin-derived compounds and an increase in aromatic compound contributions with soil depth. The higher relative lignin content in surface soils likely reflects recent plant inputs from vascular tissue. Aromatic compounds are chemically more stable and persist under oxygen-limited conditions.^41–43^ Combined, these findings indicate that as soil depth increases, lignin is progressively broken down or transformed, while less bioavailable aromatic structures accumulate.

The observed trends in SOC content and composition and the soil redox state align with the expected sequential microbial use of substrates based on reaction thermodynamics. Compared to polysaccharides, phenolic and aromatic compounds are chemically more reduced on average and therefore require higher energy input to be oxidized. Under anoxic conditions, this required energy input may outweigh the energy released upon reduction of alternative TEAs, resulting in the accumulation of these compounds.^4,7^

### Microbial community composition and potential functions are linked to landscape position and soil physicochemical characteristics

Microbial community composition differed between plain and slope soils (Fig. 5), with plain soils having higher relative abundances of potential anaerobic microbial respiratory pathways, in agreement with our second hypothesis. These higher abundances may reflect seasonal moisture fluctuations and nutrient-rich conditions. Recurrent anoxic windows were associated with a rich assemblage of anaerobic microbial metabolisms, including the potential reduction of nitrate, iron, manganese, and sulfate, consistent with ongoing SOC turnover in the riparian corridor.^10^ We found higher abundance of taxa commonly associated with anaerobic respiration at soil depth in plain soils, including members of the *Chloroflexota* or *Desulfobacterota* phyla,^44^ in line with the redox stratification inferred from EDC–EAC profiles. Conversely, slope soils were dominated by phyla such as *Verrucomicrobiota*, *Thermoproteota* and *Dormibacterota*. These soils were well-drained and oxygen-rich and, in concert with lateral inputs of carbohydrate-rich litter, were associated with high-energy-yielding aerobic microbial respiration pathways. Canonical methanogenesis genes were not detected in the recovered MAGs from either plain or slope soils; however, non-detection in our metagenomic dataset does not necessarily indicate ecological absence. Methanogens may occur at low abundance, be restricted to deeper and more persistently waterlogged horizons, or remain in short and unbinned contigs that were not recovered during assembly and binning. The sequencing depth of shotgun metagenomics may also have been insufficient to recover rare methanogenesis genes, which can be difficult to detect in complex soil communities. By contrast, low-abundance methane oxidation genes were detected, suggesting that methane cycling may still occur in these soils even though methanogenesis potential was not recovered in the MAG dataset.^15^ In addition, Group 3 and Group 1 NiFe hydrogenases were more highly represented in plain soils (Fig. S3), consistent with greater redox flexibility and the capacity of these communities to alternate between fermentative and respiratory strategies as oxygen availability fluctuates.^45^

Microbial community composition aligned with the observed differences in SOC composition across landscape positions (Fig. 5). Plain soils were associated with phenol-rich litter derived from hydrophilic grasses and sedges, consistent with microbial communities adapted to the partial degradation of these compounds under oxygen-limited conditions.^45,46^ The association between phenolic compounds and anoxic conditions is consistent with slow decomposition of soil organic matter, and these compounds may also have contributed to the elevated EDC observed in plain soils by providing redox-active moieties that can function as extracellular electron shuttles.^47,48^ In contrast, microbial communities in slope soils were dominated by fast-growing copiotrophs and were linked to polysaccharide-rich substrates, congruent with the higher proportion of depolymerisable carbohydrates we observed in these soils. Building on the previous discussion, the abundance of aromatic compounds increased with soil depth, whereas lignin-derived phenols decreased. Surface soil horizons were characterised by a higher relative abundance of lignin-derived compounds and by conditions consistent with microbial use of readily degradable polysaccharides and relatively rapid lignin turnover.^49,50^

Several environmental variables influenced the microbial community structure in plain and slope soils (Fig. 8). EAC, Ca content, pH and phenolic content explained most variance in microbial community composition in plain soils. Calcium has previously been shown to stabilise SOC through cation bridging with negatively charged organic surfaces, potentially restricting microbial access to SOC.^51^ Given the pH-dependence of these interactions and the propensity of alpine plain systems to experience seasonal water saturation,^52^ it is plausible that associated redox and pH fluctuations influenced microbial niche differentiation.^53^ The observed association between phenolic content and microbial community composition may also be consistent with the presence of redox-active substrates linked to microbial groups capable of utilising these substrates either as carbon sources or as electron shuttles under oxygen-limited conditions. In slope soils, microbial assemblages were more closely aligned with polysaccharide content, EDC, and clay content. While mean EDC values were relatively low, the spatial variation across samples may point to micro-heterogeneity in the distribution of redox-active substrates, which could influence microbial organisation even under well-drained conditions. Polysaccharides, derived from rapid cycling of plant litter, may provide readily accessible energy and were associated with copiotrophic lineages (notably several proteobacterial MAGs that our metagenomic analysis showed to be enriched in slope soils). Our results suggest that substrate quality, rather than the amount of SOC only, shapes microbial assemblages under well-aerated conditions.

Our findings align with emerging evidence from other alpine regions, including the Qinghai–Tibet Plateau, where microbial community composition and carbon-cycling functions have been linked to gradients in soil moisture, pH, and wetland hydrology. Similar to our plain–slope contrast, studies from Tibetan alpine wetlands and riparian ecosystems suggest that water availability and associated geochemical conditions are key regulators of the microbial functional structure.^54^ Comparable process-level patterns have also been described in permafrost soils, where metagenomic analyses indicate that redox-sensitive pathways, especially iron cycling, can strongly influence microbial carbon turnover.^55–57^ These similarities suggest that hydrology and redox state may represent general organising controls across cold-region soils, although the magnitude of their effects remains ecosystem-specific.

Our results also have implications for how alpine riparian carbon cycling may respond to future hydrological change. If climate warming lowers water tables in plain areas and increases soil oxygenation, the currently more reduced, phenol-rich SOC pool may become more susceptible to microbial decomposition as thermodynamic constraints on oxidation are alleviated. Conversely, if extreme precipitation events become more frequent on slopes, transient oxygen limitation may promote the temporary expansion of anaerobic microbial metabolisms and alter the balance between aerobic mineralisation and redox-sensitive carbon turnover. These process-based responses are consistent with the differences in soil redox state, substrate composition, and microbial functional potential observed between plain and slope soils in our study, although their magnitude will likely depend on local hydrology, vegetation, and mineralogical context.

## Conclusions

Our work shows how microbial community composition varies across landscape positions from wet plain soils to drier slope soils in alpine riparian zones. Plain soils contained three to four times more SOC than adjacent slope soils, were enriched in phenolic compounds, had higher EDC values, and harbored microbial communities with genes for nitrate, iron, manganese, and sulfate reduction—features consistent with periodic anoxia and the accumulation of SOC due to thermodynamic limitations on microbial activity. In contrast, slope soils had lower SOC contents, were not reduced, had a higher proportion of labile polysaccharides, and had microbial communities dominated by aerobic taxa. Together, these patterns demonstrate how moisture-driven redox regimes shape microbial potential and SOC composition, influencing the balance between SOC preservation and mineralization across the landscape. By comparing analogous landscape positions in two independent alpine catchments, our work provides a case study of how topographically-driven soil redox gradients govern microbial ecology.

Several open questions remain regarding the role of microbial metabolism in carbon cycling in alpine riparian soils, particularly during seasonal transitions. Microbial communities may remain active beneath the winter snowpack, but their response to the spring melt pulse of dissolved organic carbon is not well understood. Future studies applying metatranscriptomics, extracellular enzyme assays, stable isotope probing, and targeted process measurements could help resolve these relationships more directly by linking microbial identity and activity to substrate use and carbon transformation under changing redox conditions. Such insights would clarify how seasonal and topographic variability regulates organic carbon turnover and ultimately the net carbon balance of alpine catchments.

## Conflicts of interest

There are no conflicts to declare.

## Supplementary Material

EM-028-D5EM01047K-s001

## Data Availability

Soil physicochemical data in this article are available at https://doi.org/10.5281/zenodo.17710964. Metagenomic sequencing data generated in this study have been deposited in the European Nucleotide Archive https://www.ebi.ac.uk/ena/browser/view/PRJEB105115. All additional data supporting the findings of this study are provided in the article and its supplementary information (SI). Supplementary information: additional details on soil physicochemistry, the complete catalogue of pyrolysis GC-MS moieties together with an example pyrogram, element-specific electron exchanging capacities for iron, sulfur, and manganese, differential expression of metabolic pathways between plain and slope soils, and correlations between functional gene categories and taxonomic lineages. See DOI: https://doi.org/10.1039/d5em01047k.
