# Supplementary material for: Emerging investigator series: metagenomic insights into microbial controls of carbon cycling in alpine soils
Source: Environ Sci Process Impacts. 2026 Apr 28;28(6):1692–703. doi: 10.1039/d5em01047k (PMC13175029; doi:10.1039/d5em01047k)
Supplement: EM-028-D5EM01047K-s001 [file EM-028-D5EM01047K-s001.pdf]

Supplementary Information (SI) for Environmental Science: Processes & Impacts.
This journal is © The Royal Society of Chemistry 2026

Supplementary Material

for

Metagenomic Insights Into Microbial Controls of Carbon Cycling in Alpine Soils

Kristina Bright[1], Bence Dienes[1], Bart van Dongen[2], Ilya Strashnov[2], Xingguo Han[3], Meret Aeppli[1*]

[1]Soil Biogeochemistry laboratory (SOIL), Swiss Federal Institute of Technology Lausanne (EPFL), Sion, Switzerland

[2]Department of Earth and Environmental Sciences, University of Manchester, Manchester, United Kingdom

[3]Swiss Federal Institute for Forest, Snow and Landscape Research (WSL), Birmensdorf, Switzerland

[*] correspondence: meret.aeppli@epfl.ch, +41 21 693 72 79

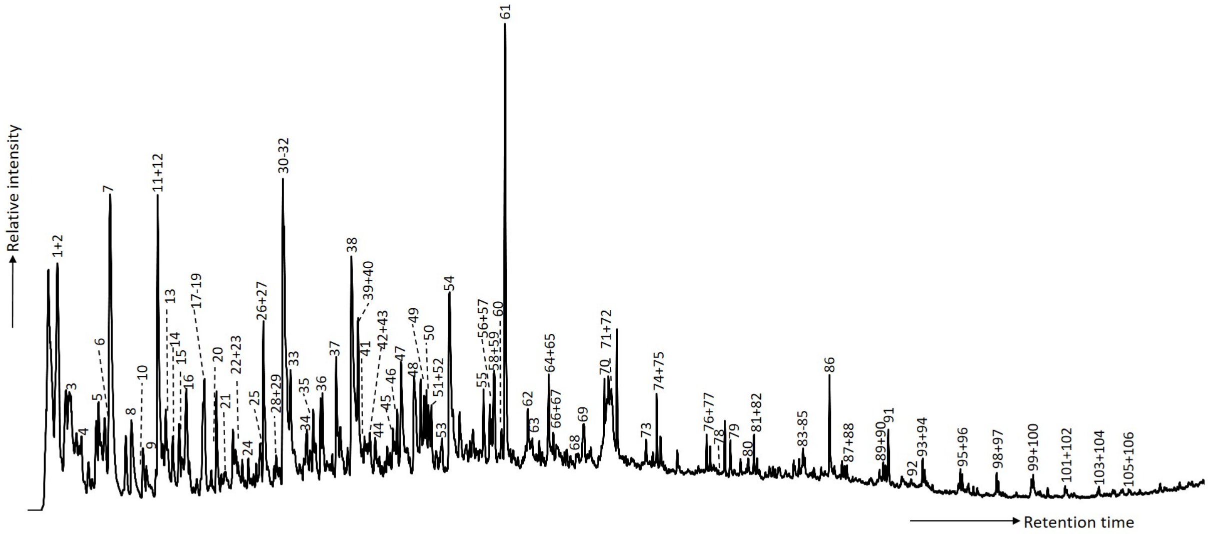

Figure S1: Example pyrogramm from pyrolysis gas chromatography–mass spectrometry analysis.

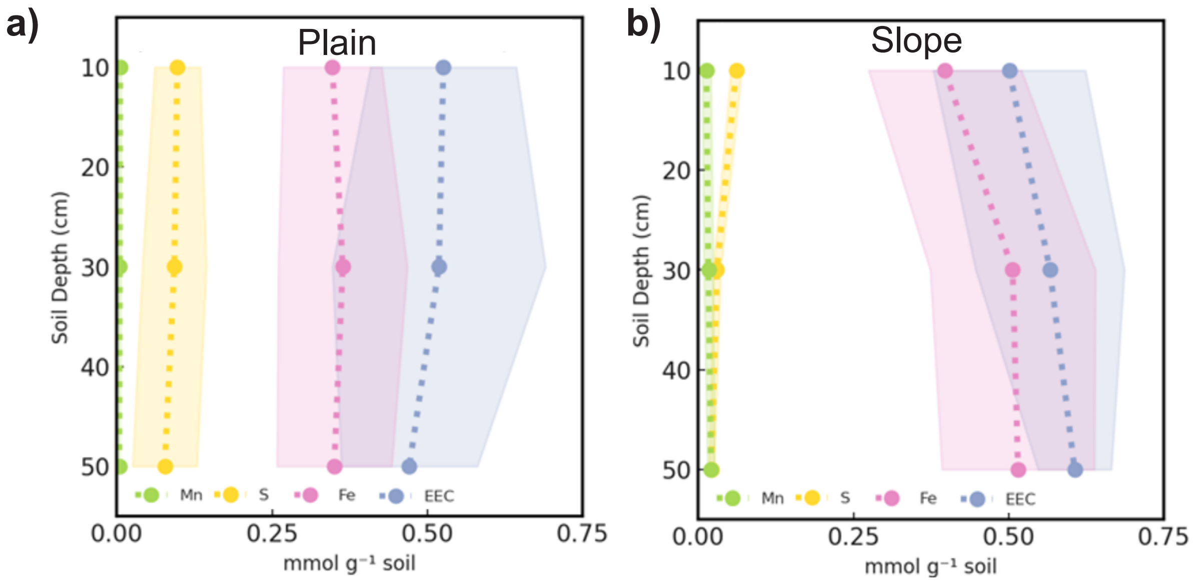

Figure S2: Electron exchanging capacity (EEC) and exchange capacities for iron, sulfur, and manganese. EEC values were determined from the sum of electron donating and accepting capacity. Element-specific exchange capacities were calculated from elemental concentrations, assuming one electron exchange per atom. Shaded areas represent the standard error of the mean. Each data point reflects the average of five soil samples.

Table S1: Soil physicochemical properties of the studied samples, including sampling depth (A = 0-10 cm, B = 10-30 cm, C = 30-50 cm), geographic coordinates, total C and N contents, texture fractions (clay, silt, sand), pH, and total Fe, Mn, S and Ca concentrations.

**Name**	**Depth**	**X coordinate**	**Y coordinate**	**C (%)**	**N (%)**	**Clay (%)**	**Silt (%)**	**Sand (%)**	**pH**	**Fe (µg/g)**	**Mn (µg/g)**	**S (µg/g)**	**Ca (µg/g)**
BF21	A	2664657	1136886	0.85	0.05	0.99	27.41	71.59	7.12	22681.88	453.64	447.31	52168.33
BF21	B	2664657	1136886	1.97	0.12	1.50	41.86	56.64	7.28	26426.30	487.16	568.58	32066.74
BF21	C	2664657	1136886	0.46	0.03	0.76	20.26	78.98	6.91	24476.06	609.95	350.95	45242.11
BF26	A	2664636	1136854	1.05	0.05	1.41	38.95	59.63	7.54	26888.50	429.49	476.00	24799.30
BF26	B	2664636	1136854	0.46	0.00	0.76	23.94	75.31	7.51	29581.46	536.06	353.19	13953.14
BF26	C	2664636	1136854	0.18	0.00	0.99	24.72	74.30	7.79	23723.51	561.49	118.67	24174.14
BS18	A	2664534	1137140	4.39	0.29	2.92	29.40	67.69	4.85	26674.56	522.83	1025.16	7360.88
BS18	B	2664534	1137140	2.83	0.17	3.52	30.20	66.27	5.02	29746.87	919.81	724.24	7098.25
BS18	C	2664534	1137140	1.68	0.10	4.95	36.56	58.49	5.22	31243.00	1293.76	512.53	6835.10
BS21	A	2664590	1137072	3.81	0.27	1.96	26.52	71.53	5.21	20716.48	627.96	1088.37	7492.29
BS21	B	2664590	1137072	2.70	0.21	3.39	34.58	62.03	5.02	25369.96	727.88	965.27	6729.88
RF20	A	2605594	1116454	15.77	1.12	3.65	60.92	35.43	6.11	32182.22	2536.16	2882.33	10888.34
RF20	B	2605594	1116454	3.38	0.26	3.14	76.22	20.64	6.52	30217.41	328.05	1541.43	5291.61
RF20	C	2605594	1116454	1.13	0.09	3.97	69.80	26.22	6.63	41011.59	263.59	1675.46	4032.77
RF24	A	2605579	1116699	7.33	0.57	3.71	80.98	15.32	5.59	42917.84	354.12	955.65	5006.22
RF24	B	2605579	1116699	1.39	0.14	3.13	67.92	28.95	6.32	33530.86	426.83	606.04	3629.57
RF24	C	2605579	1116699	0.53	0.04	3.85	56.51	39.64	7.01	46108.15	432.28	239.52	2336.01
RS13	A	2605229	1116612	3.58	0.28	3.40	50.91	45.69	6.85	35590.51	299.27	1048.57	4279.37
RS13	B	2605229	1116612	2.49	0.24	4.87	59.32	35.81	6.89	41163.72	2543.83	916.54	5306.71
RS13	C	2605229	1116612	1.97	0.20	4.53	60.27	35.20	6.96	39876.05	992.94	1005.23	4996.69
RS22	A	2605273	1116750	4.68	0.32	5.35	55.86	38.79	5.23	37034.96	848.14	994.31	2835.53
RS22	B	2605273	1116750	1.49	0.12	3.88	47.78	48.34	6.47	34777.78	838.54	442.37	3028.28

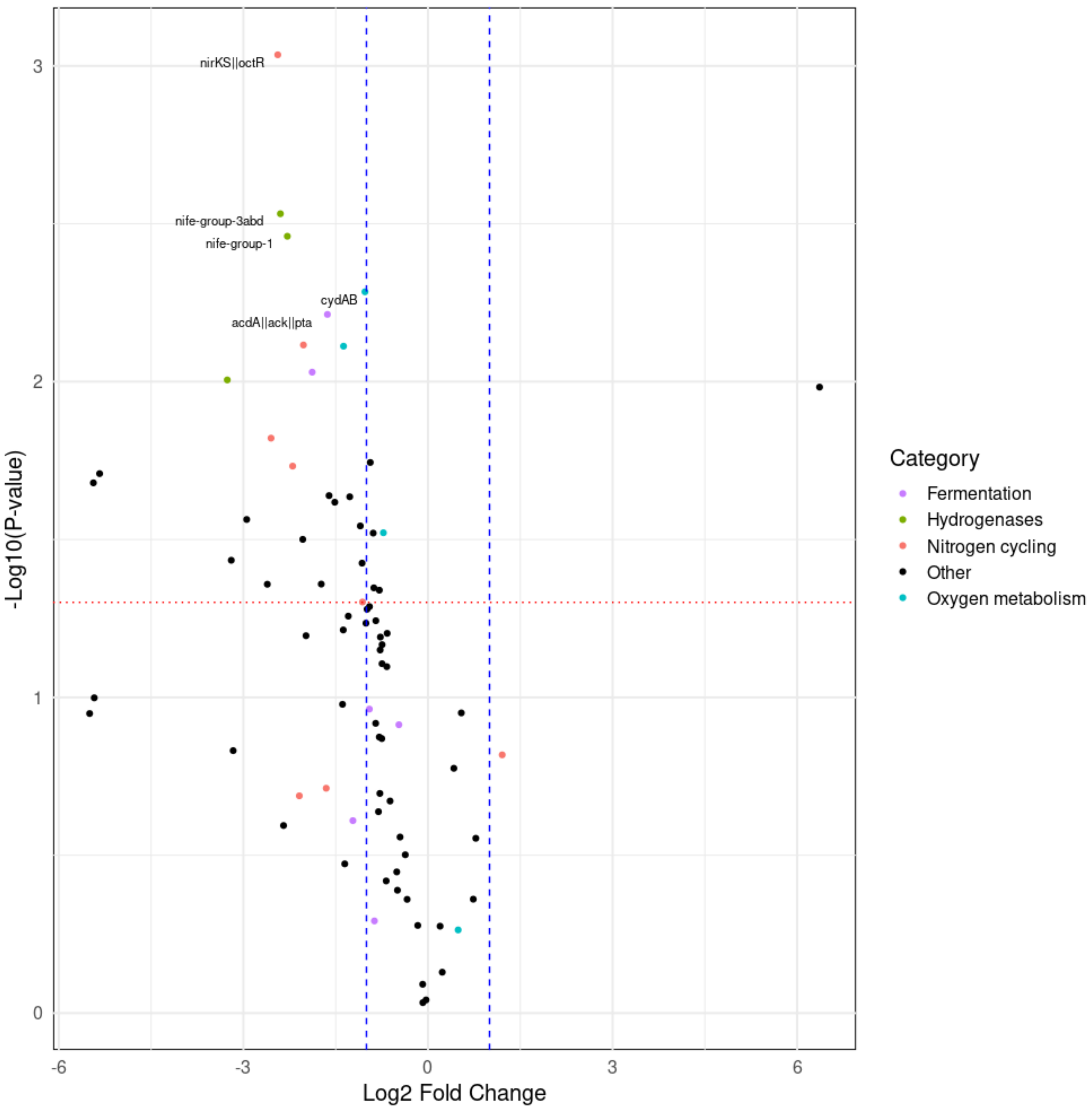

Figure S3: Volcano plot showing differential gene expression for metabolic pathways between plain (left) and slope soils (right).

Table S2: List of pyrolysis gas chromatography–mass spectrometry moieties found in the studied soils, containing peak number (as related to Figure S1), retention time, compound class, molecular weight and masses used quantification. [a] ncc = nitrogen containing compounds

**Pyrolysis moiety**	**Peak #**	**Retention time (min)**	**Compound class[a]**	**Molecular weight**	**Masses**
2-methylfurnan	1	2.4	polysaccharides	82	53+82
acetic acid	2	2.4	polysaccharides	60	60
benzene	3	3.0	aromatics	78	77+78
(1H)-pyrrole, dimethyl	4	3.4	ncc	96	95+96
Pyridine	5	4.2	ncc	79	52+79
Pyrrole	6	4.5	ncc	67	67
toluene	7	4.7	aromatics	92	92+91
(2H)-furan-3-one	8	5.7	polysaccharides	84	54+84
3 furaldehyde	9	6.2	polysaccharides	96	95+96
methylpyridine	10	6.4	ncc	93	66+93
cyclopenten-1-one	11	6.8	polysaccharides	82	82+54
Furfural	12	6.9	polysaccharides	96	95+96
methyl-1H-pyrrole	13	7.2	ncc	81	80+81
methyl-1H-pyrrole	14	7.5	ncc	81	80+81
C2 bezene (xylene)	15	7.8	aromatics	106	106+91
C2 bezene (xylene)	16	8.1	aromatics	106	106+91
styrene	17	8.9	aromatics	104	104+78
C2 bezene (xylene)	18	9.0	aromatics	106	106+91
C9 alkene	19	9.0	lipids	126	55+69
C9 alkane	20	9.3	lipids	128	57+71
acetylfuran	21	9.7	polysaccharides	110	110+95

Continue on the next page

Table S2: (cont.)

**Pyrolysis moiety**	**Peak #**	**Retention time (min)**	**Compound class**[a]	**Molecular weight**	**Masses**
2hydroxy-2-cyclopenten-1-one	22	10.3	polysaccharides	98	98+55
dimethylpyridine	23	10.5	ncc	107	106+107
propylbenzene	24	11.2	aromatics	120	120+91
C3 benzene	25	11.5	aromatics	120	105+120
5 methyl fufural	26	11.6	polysaccharides	110	110+109
C3 benzene	27	11.7	aromatics	120	105+120
C3 benzene	28	12.1	aromatics	120	105+120
benzonitrile	29	12.3	ncc	103	76+103
Phenol	30	12.5	phenols	94	94+66
c10 alkene	31	12.5	lipids	140	55+69
C3 benzene	32	12.6	aromatics	120	105+120
C10 alkane	33	12.9	lipids	142	57+71
C3 benzene	34	13.6	aromatics	120	105+120
3-hydroxy-2-methyl-2-cyclopenten-1-one	35	13.9	polysaccharides	112	112
Indene	36	14.3	aromatics	116	116+115
methylphenol	37	14.9	phenols	108	107+108
methylphenol	38	15.6	phenols	108	107+108
4-methoxyphenol (guaicol)	39	15.9	lignins	124	109+124
c11 alkene	40	15.9	lipids	154	55+69
C11 alkane	41	16.2	lipids	156	57+71
methylbenzofuran	42	16.3	polysaccharides	132	132+131

Continue on the next page

Table S2: (cont.)

**Pyrolysis moiety**	**Peak #**	**Retention time (min)**	**Compound class**^{a}	**Molecular weight**	**Masses**
methylbenzofuran	43	16.4	polysaccharides	132	132+131
maltol	44	16.8	polysaccharides	126	126
benzylnitrile	45	17.5	ncc	117	90+117
3methyl 1H-indene	46	17.6	aromatics	130	130+115
dimethyl/ethylphenol	47	17.8	phenols	122	107+122
dimethyl/ethylphenol	48	18.4	phenols	122	107+122
naphthalene	49	18.7	aromatics	128	128
c12 alkene	50	19.0	lipids	168	55+69
4-methylguiaacol (creosol)	51	19.1	lignins	138	123+138
C12 alkane	52	19.2	lipids	170	57+71
4,7-dimethylbenzofuran	53	19.7	polysaccharides	146	145+146
4-vinylphenol	54	20.1	phenols	120	120+91
4-ethylguaiacol	55	21.5	lignins	152	137+152
c13 alkene	56	21.8	lipids	182	55+69
methyl naphthalene	57	21.9	aromatics	142	142+141
Indole	58	22.0	ncc	117	90+117
C13 alkane	59	22.0	lipids	184	57+71
methyl naphthalene	60	22.4	aromatics	142	142+141
4-vinylguaiacol	61	22.5	lignins	150	135+150
2,6-dimethoxyphenol (syringol)	62	23.6	lignins	154	139+154
4-Propenylguaiacol	63	23.7	lignins	164	164+149

Continue on the next page

Table S2: (cont.)

**Pyrolysis moiety**	**Peak #**	**Retention time (min)**	**Compound class**[a]	**Molecular weight**	**Masses**
methyl indole	64	24.5	ncc	131	131+130
c14 alkene	65	24.5	lipids	196	55+69
C14 alkane	66	24.7	lipids	198	57+71
4-formylguaicol (Vanillin)	67	24.9	lignins	152	151+152
4-methylsyringol	68	26.0	lignins	168	153+168
trans-4-(2-propenyl)guaiacol (eugenol)	69	26.0	lignins	164	164+149
C15 alkene	70	27.0	lipids	210	55+69
levoglucosan	71	27.3	polysaccharides	162	73+60
C15 alkane	72	27.2	lipids	212	57+71
4-vinylsyringol	73	28.9	lignins	180	165+180
C16 alkene	74	29.3	lipids	224	55+69
C16 alkane	75	29.5	lipids	226	57+71
C17 alkene	76	31.6	lipids	238	55+69
C17 alkane	77	31.8	lipids	240	57+71
diketodipyrrole	78	32.0	NCC	186	93+186
4-acetylsyringol	79	32.7	lignins	196	181+196
phenanthrene	80	33.5	aromatics	178	178
C18 alkene	81	33.7	lipids	252	55+69
C18 alkane	82	33.9	lipids	254	57+71
C19 alkene	83	35.8	lipids	266	55+69
C19 alkane	84	35.9	lipids	268	57+71

Continue on the next page

Table S2: (cont.)

**Pyrolysis moiety**	**Peak #**	**Retention time (min)**	**Compound class**[a]	**Molecular weight**	**Masses**
C17 methylketone	85	36.0	lipids	254	58+59
C16 fatty acid	86	37.1	lipids	256	60+73
C20 alkene	87	37.7	lipids	280	55+69
C20 alkane	88	37.8	lipids	282	57+71
C21 alkene	89	39.5	lipids	294	55+69
C21 alkane	90	39.7	lipids	296	57+71
C19 methylketone	91	39.8	lipids	282	58+59
C18 fatty acid	92	40.8	lipids	284	60+73
C22 alkene	93	41.3	lipids	308	55+69
C22 alkane	94	41.4	lipids	310	57+71
C23 alkene	95	43.0	lipids	322	55+69
C23 alkane	96	43.1	lipids	324	57+71
C24 alkene	97	44.7	lipids	336	55+69
C24 alkane	98	44.8	lipids	338	57+71
C25 alkene	99	46.2	lipids	350	55+69
C25 alkane	100	46.3	lipids	352	57+71
C26 alkene	101	47.8	lipids	364	55+69
C26 alkane	102	47.8	lipids	366	57+71
C27 alkene	103	49.2	lipids	378	55+69
C27 alkane	104	49.3	lipids	380	57+71
C28 alkane	105	50.7	lipids	394	57+71
C29 alkane	106	52.0	lipids	408	57+71

Table S3: Spearman correlations between functional gene categories and taxonomic lineages identified in metagenomic data. Lineages are grouped by taxonomic rank: p = phylum, c = class, o = order. Only statistically significant associations ($p < 0.05$) are shown.

**Taxon Lineage**	**Potential Function**	**Correlation Estimate**	**P-Value**
p__Chloroflexota<br>c__Anaerolineae<br>o__Anaerolineales	Nitrate reduction	0.598	0.00535
p__Chloroflexota<br>c__Dehalococcoidia<br>o__DSTF029	Nitrate reduction	0.560	0.01025
p__Chloroflexota<br>c__Dehalococcoidia<br>o__SM23-31	Nitrate reduction	0.372	0.01802
p__Acidobacteriota<br>c__Thermoanaerobaculia<br>o__Thermoanaerobaculales	Fe/Mn reduction	0.786	9.83e-14
p__Acidobacteriota<br>c__Acidobacteriae<br>o__Acidobacterales	Fe/Mn reduction	0.738	0.000202
p__Acidobacteriota<br>c__Blastocatellia<br>o__Pyrinomonadales	Fe/Mn reduction	0.734	0.000228
p__Desulfobacterota<br>c__Desulfuromonadia<br>o__Geobacterales	Sulfate reduction	0.400	0.01051
p__Desulfobacterota<br>c__BSN033<br>o__BSN033	Sulfate reduction	0.466	0.03830
p__Desulfobacterota<br>c__DSM-4660<br>o__Desulfatiales	Sulfate reduction	0.457	0.04254
